# Novel combination immunotherapy for pancreatic cancer: potent anti‐tumor effects with CD40 agonist and interleukin‐15 treatment

**DOI:** 10.1002/cti2.1165

**Published:** 2020-08-15

**Authors:** Jonas RM Van Audenaerde, Elly Marcq, Bianca von Scheidt, Ashleigh S Davey, Amanda J Oliver, Jorrit De Waele, Delphine Quatannens, Jinthe Van Loenhout, Patrick Pauwels, Geert Roeyen, Filip Lardon, Clare Y Slaney, Marc Peeters, Michael H Kershaw, Phillip K Darcy, Evelien LJM Smits

**Affiliations:** ^1^ Center for Oncological Research (CORE) Integrated Personalized & Precision Oncology Network (IPPON) University of Antwerp Wilrijk Belgium; ^2^ Cancer Immunotherapy and Immune Innovation Laboratory Peter MacCallum Cancer Centre Melbourne VIC Australia; ^3^ Department of Pathology Antwerp University Hospital Edegem Belgium; ^4^ Department of Hepatobiliary, Endocrine and Transplantation Surgery Antwerp University Hospital Edegem Belgium; ^5^ Sir Peter MacCallum Department of Oncology The University of Melbourne Parkville VIC Australia; ^6^ Department of Oncology and Multidisciplinary Oncological Centre Antwerp Antwerp University Hospital Edegem Belgium; ^7^ Center for Cell Therapy and Regenerative Medicine Antwerp University Hospital Edegem Belgium

**Keywords:** CD40 agonist, combination immunotherapy, interleukin‐15, natural killer cells, pancreatic cancer, T cells

## Abstract

**Objectives:**

With the poorest 5‐year survival of all cancers, improving treatment for pancreatic cancer is one of the biggest challenges in cancer research. We sought to explore the potential of combining both priming and activation of the immune system. To achieve this, we combined a CD40 agonist with interleukin‐15 and tested its potential in pancreatic cancer.

**Methods:**

Response to this combination regimen was assessed in pancreatic ductal adenocarcinoma mouse models, and a thorough analysis of the tumor microenvironment was performed.

**Results:**

We demonstrated profound reduction in tumor growth and increased survival of mice with the majority of mice being cured when both agents were combined, including an unprecedented 8‐fold dose reduction of CD40 agonist without losing any efficacy. RNAseq analysis showed involvement of natural killer (NK) cell‐ and T‐cell‐mediated anti‐tumor responses and the importance of antigen‐presenting cell pathways. This combination resulted in enhanced infiltration of tumors by both T cells and NK cells, as well as a striking increase in the ratio of CD8^+^ T cells over Tregs. We also observed a significant increase in numbers of dendritic cells (DCs) in tumor‐draining lymph nodes, particularly CD103^+^ DCs with cross‐presentation potential. A critical role for CD8^+^ T cells and involvement of NK cells in the anti‐tumor effect was highlighted. Importantly, strong immune memory was established, with an increase in memory CD8^+^ T cells only when both interleukin‐15 and the CD40 agonist were combined.

**Conclusion:**

These novel preclinical data support initiation of a first‐in‐human clinical trial with this combination immunotherapy strategy in pancreatic cancer.

## Graphical abstract

With the poorest 5‐year survival of all cancers, improving treatment for pancreatic cancer is one of the biggest challenges in cancer research. We demonstrate in this study significant tumor reduction and prolonged survival, including complete cures, in two mouse models of pancreatic cancer. This was mediated by a significant increase of natural killer and T cells in the tumors as well as a striking increase in the ratio of CD8^+^ T cells over Tregs. These data support initiation of a first‐in‐human clinical trial with this combination immunotherapy strategy in pancreatic cancer.

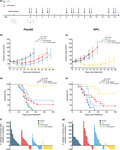

## Introduction

Pancreatic ductal adenocarcinoma (PDAC) is the third most lethal cancer worldwide with a 5‐year survival of barely 8%.^1^, ^2^ It is even projected to become the second leading cause of cancer‐related death by 2030.^3^ To date, it remains one of the most aggressive and challenging malignancies because of a complex tumor microenvironment including a strong desmoplastic reaction,^4^ low immunogenicity^5^, ^6^ and a molecular signature in favor of the tumor, driven by loss of multiple tumor suppressor genes.^7^ Despite all efforts made in the past, almost no improvement in survival has been achieved, rendering PDAC a disease which represents the very definition of an urgent unmet need for novel therapeutic approaches to finally improve the outcome of patients.

About 85% of patients are not eligible for curative surgical resection because of locally advanced or metastatic disease at the time of diagnosis. Hence, patients are treated with either FOLFIRINOX or gemcitabine/nab‐paclitaxel depending on their physical fitness. However, these treatments are associated with major toxicity issues and have limited impact.^8^, ^9^ New promising approaches that are successful in other cancer types, such as anti‐PD‐1 and anti‐CTLA‐4, have shown little improvement over treatment with gemcitabine.^10^, ^11^ This highlights the need for other novel compounds to enter the battle arena of PDAC.

In this context, the use of CD40 agonists in PDAC has been explored during the past decade, both as a single modality^12^, ^13^ and in combination with current first‐line treatments^14^, ^15^ or other compounds such as checkpoint inhibitors, PD‐1 and CTLA‐4.^16^ Anti‐tumor responses have been reported in both mice and humans, and there is a general consensus that CD40 agonist therapy provides the necessary immune‐priming signals to convert the immunogenic cold tumor microenvironment to a desirable hot inflammatory microenvironment.^17^ In addition, all studies demonstrated that combination therapy involving CD40 agonists provided more potent results in terms of tumor growth suppression and extended survival.^15^, ^16^ These data support further investigation of combination approaches with other promising candidates to unlock the full potential of CD40 agonist therapy. Since interleukin (IL‐)15 is an essential cytokine for activation and maintenance of immune effector cells, there is a strong rationale for combining immune‐priming agents with this molecule.

We have previously shown *in vitro* that IL‐15‐stimulated natural killer (NK) cells can kill both PDAC tumor cells and stromal pancreatic stellate cells which are responsible for the poor response to treatment.^18^ IL‐15 is a versatile cytokine which stimulates both T‐cell proliferation and generation of cytotoxic T lymphocytes, as well as activation and expansion of natural killer (NK) cells. Furthermore, it has the capability to induce CD8^+^ T‐cell memory cells, thereby playing a crucial role in maintaining long‐lasting immune responses to malignant cells and possible prevention of tumor relapse.^19^, ^20^, ^21^ All these features render IL‐15 a highly attractive cancer immunotherapeutic as confirmed by its high rank in the NCI's top 20 immunotherapeutic drugs with the greatest potential for broad usage in cancer therapy.^22^ Moreover, IL‐15 needs to be trans‐presented by the IL‐15Rα on dendritic cells (DCs) to its target to be effective.^20^, ^23^ Since it has been demonstrated that CD40 agonists also increase the expression of IL‐15Rα on DCs, we hypothesised that combining both agents might result in enhanced immune activation and increased anti‐tumor effects.^24^

In this article, we show for the first time in mice with pancreatic tumors that when CD40 agonist antibody and IL‐15 are combined, they exhibit synergistic effects in terms of enhanced anti‐tumor efficacy resulting in profound increases in long‐term survival with complete cure in the majority of cases. Moreover, an unprecedented striking dose reduction of CD40 agonist was possible by the addition of IL‐15. The anti‐tumor effect was found to be mediated predominantly by CD8^+^ T cells and NK cells, supported by increased amounts of CD103^+^ dendritic cells (DC) with unique cross‐presenting capacity. The infiltration of tumors by both cell types was commensurate with a reduction in the amount of regulatory T cells. These novel translational preclinical data provide a solid rationale to initiate a clinical trial investigating this novel immunotherapy combination strategy for patients with one of the hardest to treat tumors nowadays.

## Results

### Combined IL‐15 and CD40 agonist therapy results in increased anti‐tumor efficacy *in vivo*

**Figure 1:**
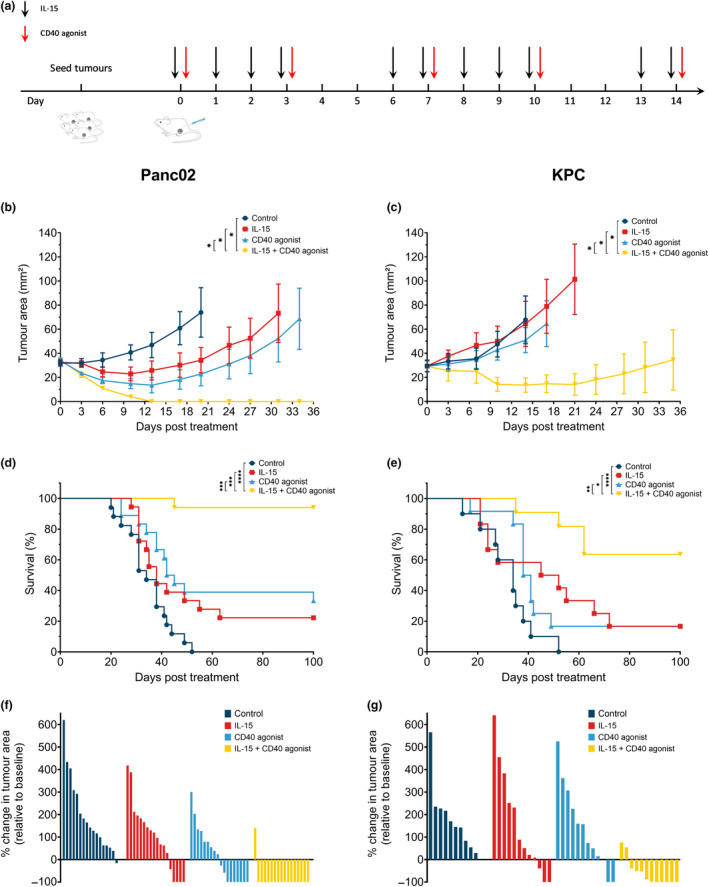
Combining IL‐15 with a CD40 agonist results in decreased tumor volume and increased survival. C57BL/6j mice were injected with either 0.5 × 10^6^ Panc02 or KPC cells subcutaneously. When tumors reached a size of 25–35 mm^2^, mice were randomised and treated with isotype control, IL‐15, CD40 agonist or IL‐15 + CD40. **(a)** Treatment scheme showing timing of dosing is indicated for IL‐15 (2.5 µg) with black arrows and for CD40 agonist or the corresponding isotype with red arrows (five doses of 12.5 µg for Panc02 or first dose 200 µg and consecutive four doses 100 µg for KPC). **(b, c)** Tumor growth kinetics are depicted [*n* = 5 or 6 mice per group, representative data of 3 (Panc02) or 2 (KPC) independent experiments]. One‐way ANOVA with Bonferroni *post hoc*. **(d, e)** Survival of Panc02 (*n* = 17) and KPC (*n* = 11) mice treated as indicated. Pooled data of 3 (Panc02) or 2 (KPC) independent experiments. Survival was determined by tumor size reaching 150 mm^2^. Log‐rank test. **(f, g)** Waterfall plots showing the % change in tumor area relative to baseline after 34 days (Panc02, *n* = 17) or 35 days (KPC, *n* = 11). Pooled data of 3 (Panc02) or 2 (KPC) independent experiments. All data represent mean ± SEM. *P ˂ 0.05; **P ≤ 0.01; ***P ≤ 0.001; ****P ≤ 0.0001.

We sought to investigate whether the combined treatment of IL‐15 and a CD40 agonist antibody may lead to augmented anti‐tumor responses in PDAC. To investigate this, C57BL/6J mice bearing either Panc02 or KPC tumors were treated with IL‐15 and/or a CD40 agonist antibody delivered intraperitoneally when tumors reached a size of 25–35 mm^2^ (Figure 1a). We demonstrated that the combination of these agents resulted in decreased tumor volumes and increased survival of mice in both the Panc02 and KPC tumor models. In the first tumor model, Panc02, we observed a significant decrease in tumor volume when mice were treated with either single agent. However, treatment with the combination regimen resulted in a further significant reduction of tumor volume, and a significant increase in survival with 16 out of 17 mice being completely tumor free (Figure 1b, d, and f and Supplementary figure 1A, C, E and G). In the second tumor model, KPC, we observed very similar results with reduced tumor volumes and increased survival of mice with the combination being significantly better than untreated control or either single‐agent therapy. In this experiment, seven out of 11 mice were completely tumor free following combination treatment (Figure 1c, e and g and Supplementary figure 1B, D, F and H). Remarkably, while we observed similar responses between the Panc02 and KPC tumor models, the dose of CD40 agonist used in the Panc02 model (five doses of 12.5 µg) was eight times lower than in the KPC model (first dose 200 µg with four consecutive doses of 100 µg). In summary, we show here that the combination of IL‐15 and CD40 agonist therapy has profound anti‐tumor activities and combining both agents leads to significant synergistic effects.

### Distinct gene expression profiles point towards combined immune priming and immune activation following therapy

**Figure 2:**
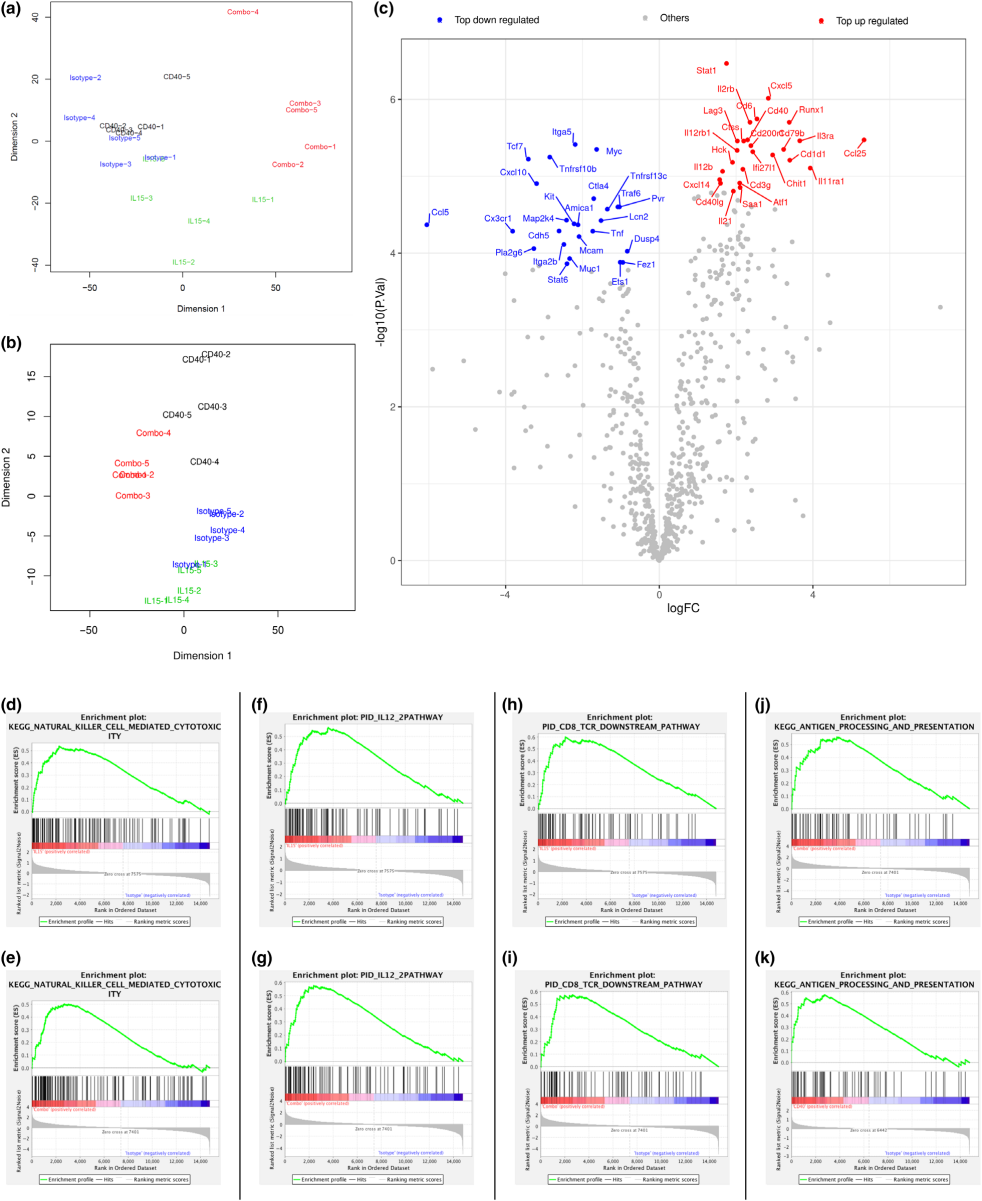
RNA profile of KPC tumors. KPC tumors were harvested on day 4 of the treatment schedule for subsequent RNA isolation and sequencing. **(a)** PCA plot showing separation of samples based on all genes. **(b)** PCA plot showing separation of samples based on immune‐related genes (Nanostring mouse immune‐related genes). **(c)** Volcano plot showing significantly differentially expressed immune‐related genes between isotype control group and the combination group. **(d–k)** GSEA plots for differentially expressed genes are showing enrichment for genes involved in a *n* = 5 tumors/group.

To unravel the effects caused by the single agents and their combination, 3′ RNA sequencing was performed on the more resistant KPC tumors harvested on day 4 in the treatment scheme. First, principal component analysis (PCA) demonstrated a clear distinction between the isotype, IL‐15 and combination group while the CD40 agonist treatment revealed a bigger overlap with the isotype control (Figure 2a). To investigate in more detail the effect of therapy on immune‐related genes, we performed a PCA using a verified Nanostring immune‐related gene panel which demonstrated clustering of the different treatment groups (Figure 2b). The top up‐ and downregulated immune‐related genes showed that genes involved in antigen presentation, T helper 1 immune type responses and NK cell cytotoxicity were modulated (Figure 2c). To confirm this, gene set enrichment analysis (GSEA) was performed showing that gene sets involved in NK cell‐mediated cytotoxicity (Figure 2d and e and Supplementary figure 2A), the IL12/2 pathway (Figure 2f and g and Supplementary figure 2B) and the CD8 TCR downstream pathway (Figure 2h and i and Supplementary figure 2C) were clearly among the top upregulated pathways in the groups when IL‐15 was administered, while the CD40 agonist treatment strongly promoted antigen processing and presentation pathways (Figure 2j and k and Supplementary figure 2D). Importantly, these features of both IL‐15 and CD40 agonist therapy were retained in the combination group.

### CD8^+^ T cells are responsible for anti‐tumor efficacy following therapy

**Figure 3:**
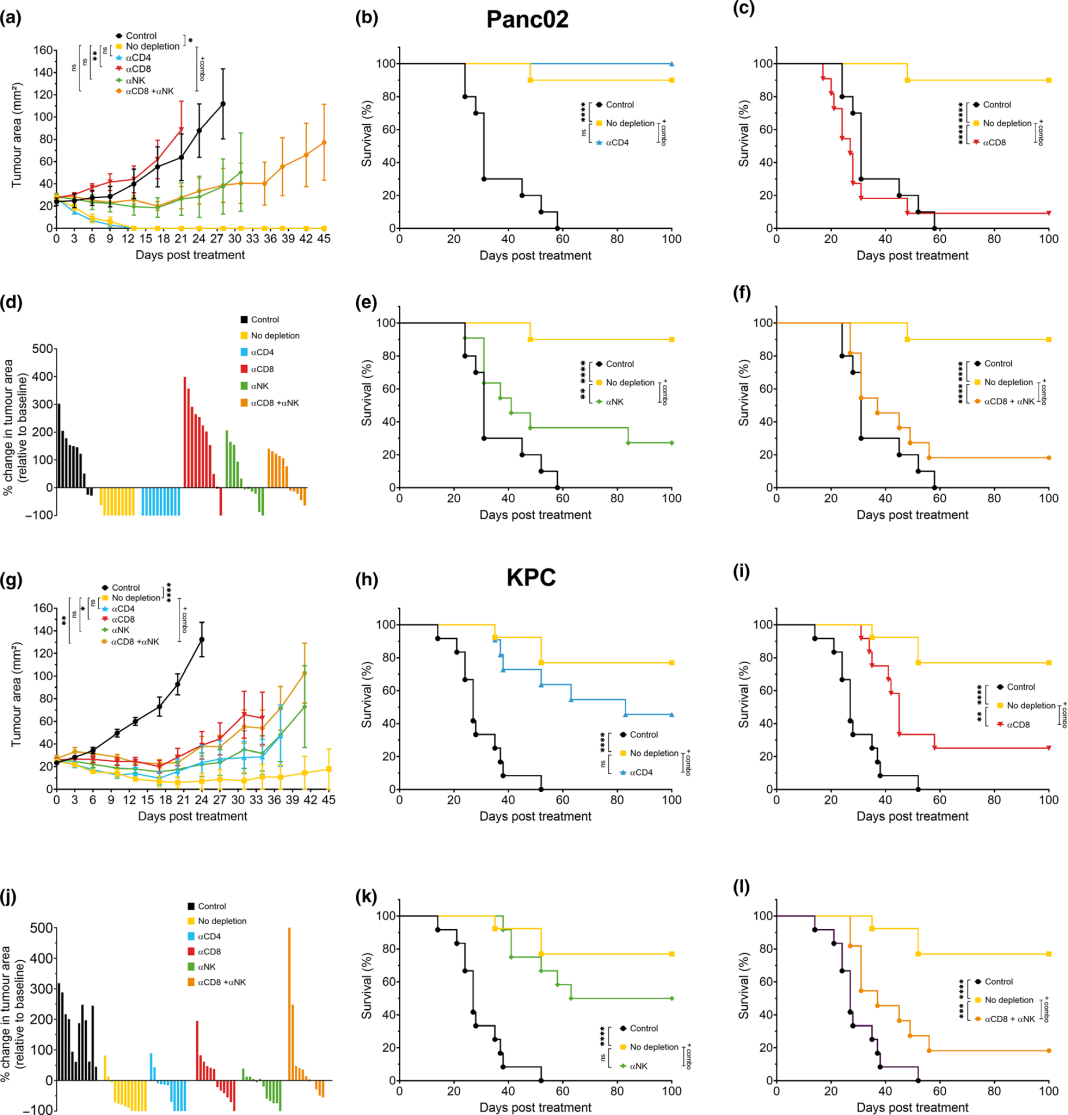
Immune cell depletion. C57BL/6j mice bearing Panc02 or KPC tumors were treated with isotype control or the IL‐15 + CD40 agonist combination regimen alone or with depleting antibodies against CD4, CD8, asialo‐GM1 (NK cell depletion). **(a, b)** Tumor growth kinetics of Panc02 or KPC tumors either non‐treated (isotype), treated with the combination regimen only (no depletion) or combination and depletion antibodies. One‐way ANOVA with Bonferroni *post hoc*. Data points represent mean ± SEM. *n* = 5–7 mice/group, representative data of two independent experiments. **(c, d)** Waterfall plots showing the % change in tumor area relative to baseline after 17 days. **(e–k)** Survival of Panc02 or KPC‐bearing mice either non‐treated (isotype), treated with the combination regiment (no depletion) or the combination and depletion antibodies against CD4 **(e, f)**, CD8 **(g, h)**, asialo‐GM1 **(i, j)** or CD8 + asialo‐GM1 **(k, l)**. Data pooled from two independent experiments with *n* = 10 or 11 (Panc02) or *n* = 11–13 (KPC). Log‐Rank test. ns *P* ≥ 0.05; *P < 0.05; **P ≤ 0.01; ***P ≤ 0.001; ****P ≤ 0.0001.

To gain more insight into the immune cells responsible for the observed anti‐tumor responses, we depleted several immune cell populations in tumor‐bearing mice in both tumor models using specific depletion antibodies. We monitored the effect the depletions had on both tumor growth kinetics and survival of mice (Figure 3a–d and Supplementary figure 3). Upon depletion of CD4^+^ T cells, we observed in both PDAC models no significant difference in survival of mice between the depleted and non‐depleted groups, indicating that CD4^+^ T cells do not play a significant role in the anti‐tumor response elicited by the IL‐15 and CD40 agonist combination treatment (Figure 3e and f). However, when CD8^+^ T and NK cells were depleted, the anti‐tumor effect of the combination treatment was significantly reduced in both tumor models (Figure 3k and l). When CD8^+^ T cells alone were depleted, there was a significant increase in tumor growth and reduced survival of mice (Figure 3g and h). However, depletion of NK cells only led to a mixed response. In the Panc02 tumor model, we clearly observed a significant decrease in survival of mice following depletion of NK cells, although interestingly there was no effect on tumor growth. In the KPC tumor model, there was a trend towards decreased survival of mice following NK cell depletion although this was not statistically significant (*P* = 0.19) (Figure 3i and j). In conclusion, these experiments demonstrated that CD8^+^ T cells were the predominant effector cell type responsible for the observed anti‐tumor effects with a clear contribution of NK cells in the Panc02 model.

### Combination therapy increases intra‐tumoral infiltration of immune cells

**Figure 4:**
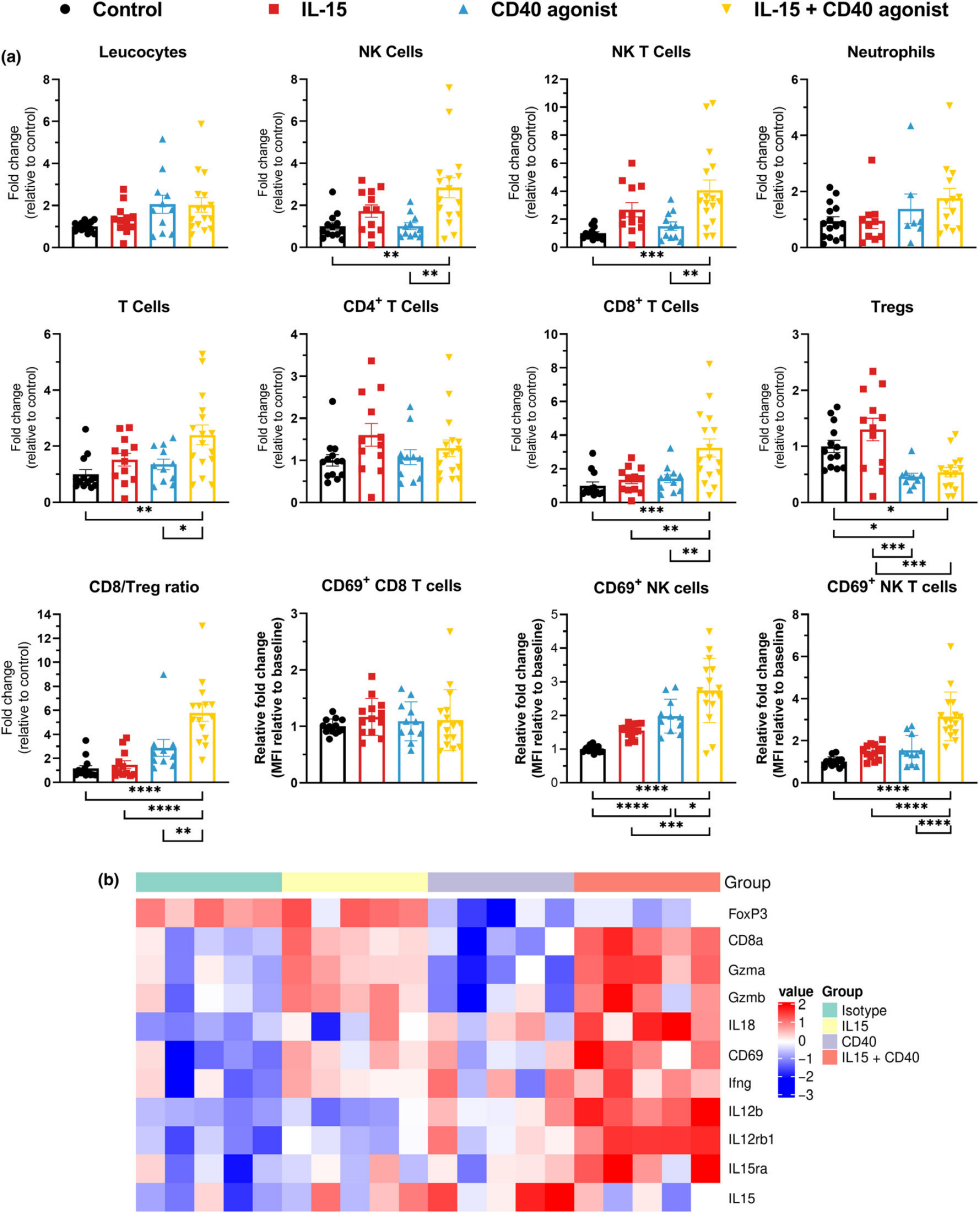
Characterisation of tumor‐infiltrating lymphocytes. C57BL/6j mice bearing KPC tumors were treated with isotype control, IL‐15, CD40 agonist or the combination of the latter. **(a)** Tumors were harvested at day 8 post‐treatment initiation. Single‐cell suspensions were acquired after enzymatic digestion for flow cytometry analysis. Immune cell populations indicated as fold change of absolute number of cells and CD69 expression (MFI) on NK, NKT and CD8^+^ T cells. Data pooled from three independent experiments, *n* = 13–16/group. One‐way ANOVA with Bonferroni. *P < 0.05; **P ≤ 0.01; ***P ≤ 0.001; ****P ≤ 0.0001. **(b)** Heatmap of gene expression of relevant genes for the quantified immune subsets. *n* = 5 tumors/group.

To further explore the anti‐cancer effects of the combination regimen, multicolour flow cytometry was utilised to monitor tumor‐infiltrating lymphocytes in the KPC tumor model (Supplementary figure 4). We observed striking differences between the different treatment arms that were tested (Figure 4a). The first observation was that the anti‐tumor effect was not due to a higher total number of infiltrating lymphocytes, but rather caused by significant differences in which immune cell subsets were present. The frequency of infiltrating NK and NKT cells was significantly higher in the combination therapy group compared to the isotype and CD40 agonist treatment groups. IL‐15 alone demonstrated an increased frequency of both cell types although this was not statistically significant compared to control‐treated mice. In terms of neutrophil numbers, although they were abundantly present, we observed no difference between the treatment groups. For T cells, we observed an increased number of total T cells following combination therapy compared to the other treatment groups. This increased number of T cells could be attributed to predominantly CD8^+^ T cells which were significantly higher in the combination therapy‐treated mice compared to all other treatment arms, while there was no difference in numbers of infiltrating CD4^+^ T cells. Interestingly, we also observed a significant decrease in numbers of regulatory T cells (Tregs) present in the tumor when CD40 agonist was part of the treatment. We also measured expression of CD69 on immune cells as an indicator of activation. We observed clear upregulation of this marker on NK and NKT cells following combination therapy, however, not on the CD8^+^ T cells. The results were confirmed by gene expression analysis of several relevant genes (Figure 4b). Here, FoxP3 transcription was indeed downregulated when Tregs were less abundant and the combination regimen demonstrated the strongest upregulation of NK(T) cell‐related genes such as granzyme A and B. The analysis revealed a strong immune activation signature as IL‐12, IL‐18, IFNγ and CD69 were upregulated. Putting these observations together, the combination treatment of CD40 agonist and IL‐15 had a more profound anti‐tumor effect as it caused a significant increase in the amount of anti‐tumor immune cells (NK, NK T and CD8^+^ T cells) compared to control or single‐agent treatment that was commensurate with a decrease in immunosuppressive Tregs, resulting in an enhanced CD8/Treg ratio within the tumors.

### Combination therapy results in increased amounts of CD103^+^ cross‐presenting DCs

**Figure 5:**
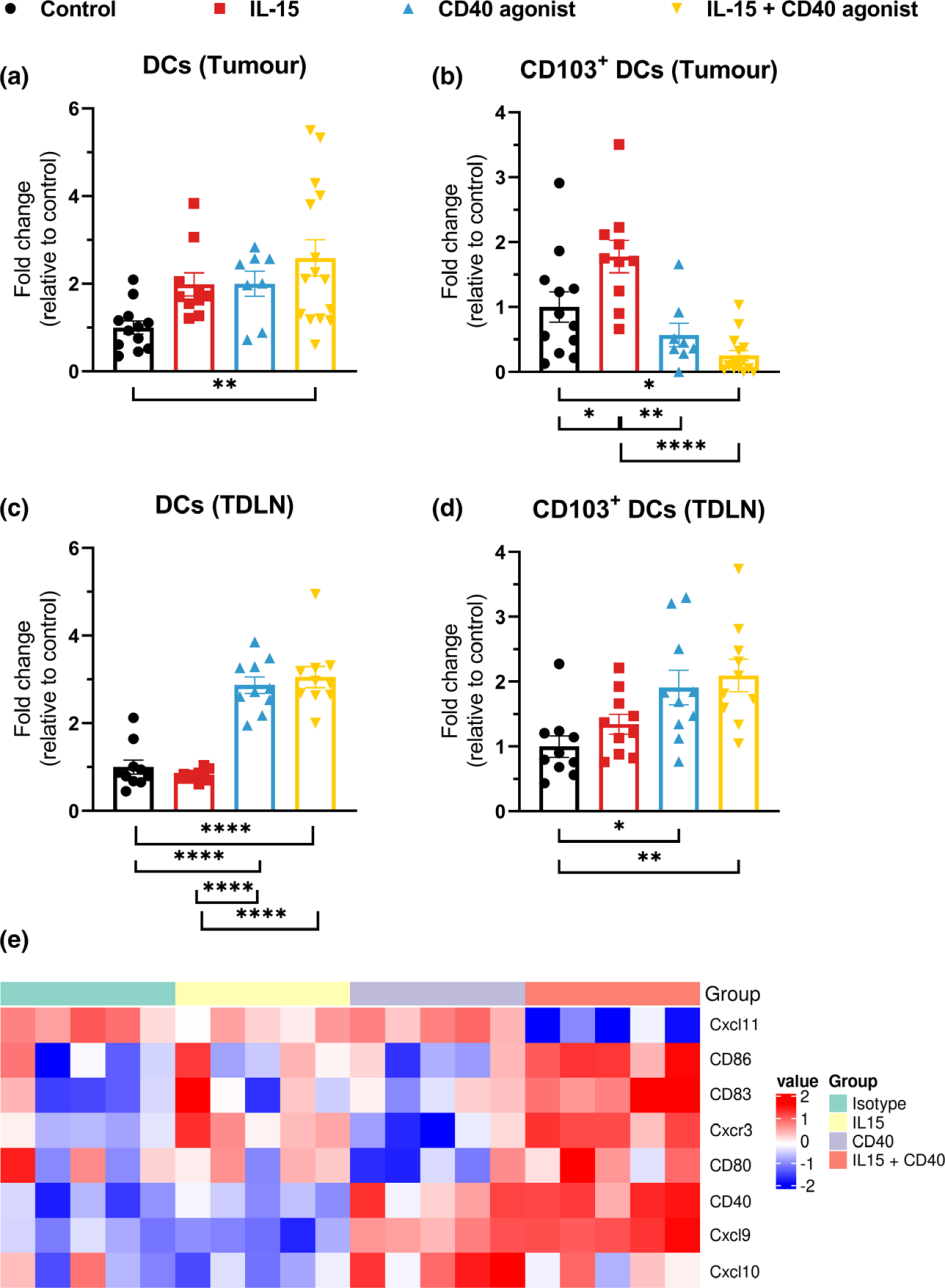
Characterisation of DCs in tumor and TDLN. C57BL/6j mice bearing KPC tumors were treated with isotype control, IL‐15, CD40 agonist or the combination of the latter. Tumors or TDLN were harvested at day 8 post‐treatment initiation. Single‐cell suspensions were acquired after enzymatic digestion for flow cytometry analysis. **(a, b)** DCs or CD103^+^ DCs in tumors. **(c, d)** DCs or CD103^+^ DCs in TDLN. Data pooled from three independent experiments, *n* = 10–16/group. One‐way ANOVA with Bonferroni. *P < 0.05; **P ≤ 0.01; ****P ≤ 0.0001. **(e)** Heatmap of gene expression of relevant genes for the quantified immune subsets. *n* = 5 tumors/group.

Dendritic cells are known to play critical roles in antigen processing and presentation and are key players in the activation of both NK and T cells. We further explored their presence in the KPC tumor model. Here, we observed a significant increase in number of DCs in the tumor, only in the combination therapy group (Figure 5a). Furthermore, the amount of CD103^+^ DCs, the subtype responsible for cross‐presentation, was determined. Here, IL‐15 caused a significant increase in the number of CD103^+^ DCs in the tumor while this was significantly lower in the groups following treatment with CD40 agonist (Figure 5b). To further investigate how the DCs behaved, we analysed the presence of these cells in the tumor‐draining lymph nodes (TDLN) and observed a 3‐fold increase in number of DCs when CD40 agonist was administered (Figure 5c). The frequency of CD103^+^ cross‐presenting DCs increased twofold under these conditions (Figure 5d), suggesting that these DCs captured antigens at the tumor site and migrated to the TDLN to activate NK and T cells. Furthermore, gene signatures showed a higher expression of CD80, CD83 and CD86 when the combination therapy was administered, indicating that likely antigen‐presenting cells like DCs are activated and mature. The increase in mRNA encoding expression of CXCR3, CXCL9 and CXCL10 (not CXCL11) as chemotactic chemokines suggests their involvement in the trafficking of anti‐tumor immune cells to the tumor site (Figure 5e).

### Induction of immune memory

**Figure 6:**
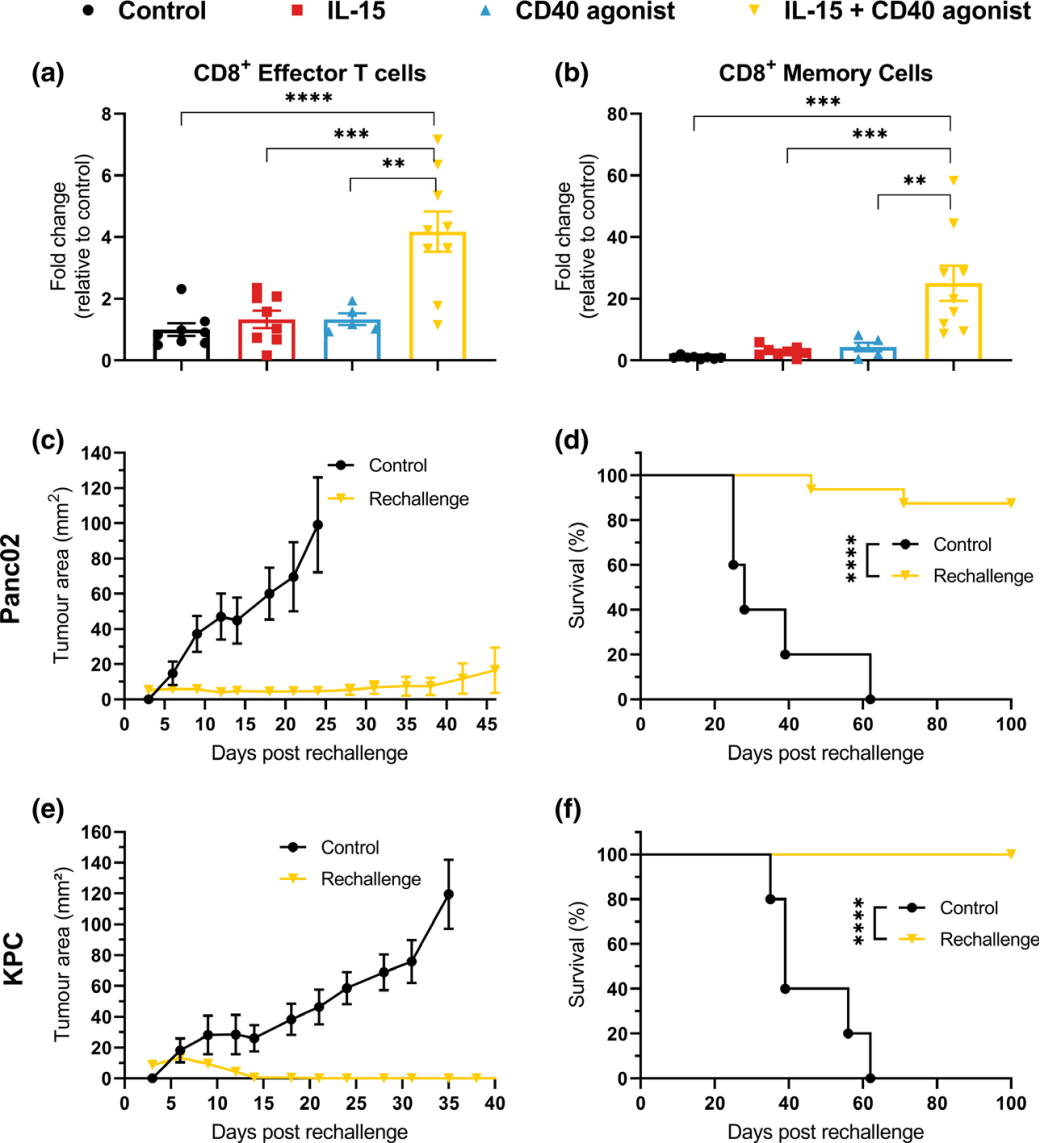
Rechallenge experiments. C57BL/6j mice cured from Panc02 or KPC tumors after treatment with IL‐15 + CD40 agonist were re‐injected with the same tumor type at the contralateral side of the abdomen. **(a, b)** Tumor kinetics and survival (log‐rank test) of mice rechallenged with Panc02 tumor cells, *n* = 16. **(c, d)** Tumor kinetics and survival of mice rechallenged with KPC tumor cells, *n* = 9. **(e, f)** Flow cytometry quantification of intra‐tumoral CD8^+^ Effector or Memory T cells of KPC tumor‐bearing mice after 8 days following treatment (*n* = 9). One‐way ANOVA with Bonferroni. **P ≤ 0.01; ***P ≤ 0.001; ****P ≤ 0.0001.

One of the goals of immunotherapy is the induction of strong immunological memory to prevent future relapse. We observed in our study an increased number of effector and central memory CD8^+^ T cells in KPC tumors when treated with the combination regimen, compared to isotype control or single arm treatments (Figure 6a and b). To investigate whether functional immune memory was induced, tumor‐free mice from the combination treatment group were rechallenged with the same tumor cell type as they were originally inoculated with. Here, we observed clear induction of immune memory in both PDAC models with 14 out of 16 mice becoming tumor free for the Panc02 tumor model and all mice becoming tumor free for the KPC tumor model compared to naïve control mice (Figure 6c–f).

## Discussion

With high resistance to current first‐line treatment and failure of numerous clinical trials, PDAC patients are in desperate need for new treatment options.^25^ There is a general consensus that CD40 agonist therapy triggers the necessary immune‐priming signalling to convert immunogenic cold tumors to hot. However, when applied in pancreatic cancer, the effects are of short duration warranting enhancement of this therapy.^12^, ^17^ IL‐15 is a powerful stimulator of NK cells and CD8^+^ T cells and induces CD44^hi^ memory T cells. This cytokine needs to be trans‐presented by the IL‐15Rα on DCs to its target to be effective.^20^, ^23^ Since it had been demonstrated that the CD40 agonist also increases the expression of IL‐15Rα on DCs, we hypothesised that combining both agents may result in enhanced immune activation and increased anti‐tumor effects. In this study, we demonstrated that IL‐15 and CD40 agonist administered alone can elicit a powerful immune response. However, when combined, these effects were strongly enhanced and importantly exceeded survival rates of CD40 agonist with gemcitabine and nab‐paclitaxel or CD40 agonist and gemcitabine and nab‐paclitaxel together with PD‐1 and CTLA‐4 blockade in similar pancreatic mouse models.^15^, ^16^ In addition when CD40 agonist was combined with anti‐angiogenic drugs blocking VEGF‐A and angiopoietin 2, survival rates did not equal the ones observed in this study.^26^ A limitation of these studies, including ours, may be that the mouse models do not completely mimic the human situation since tumor cells were injected subcutaneously. Nevertheless, CD40 agonists given as a single modality have shown beneficial effect also in human clinical trials with PDAC patients^13^, ^14^, ^27^ and our previous studies have demonstrated that upon IL‐15 stimulation, human NK cells can kill both pancreatic cancer and stromal stellate cells in an autologous human *ex vivo* setting.^18^

The potential of this combination regimen is not just limited to PDAC, since IL‐15 and CD40 agonist therapy has been tested by others in mice bearing established CT26 and MC38 colorectal tumors. The authors showed promising results albeit with less surviving mice compared to our study.^28^ This might be due to the fact that we gave in total five doses of CD40 agonist instead of four as in the other studies. Furthermore, results of other investigators using this combination therapy in a prostate cancer model TRAMP‐C2 demonstrated similar numbers of surviving mice as we found, underscoring the enormous potential of the combination approach.^24^ Of note, both colorectal cancer and prostate cancer have a significant better 5‐year overall survival of 64% and 88%, respectively, underscoring the significance of our findings in pancreatic cancer with a 5‐year survival of barely 8%.^29^, ^30^ Strikingly, in this study we also demonstrated that IL‐15 potentiates CD40 agonist treatment, causing an 8‐fold dose reduction in one of the PDAC mouse models which has not been reported so far. This important dose reduction could be of great translational importance as lower doses might significantly decrease adverse events in patients.

We observed that the combination therapy influenced several immune cell types in favor of increased anti‐tumor efficacy. As also observed by others in prostate cancer, there was an increase in number of intra‐tumoral effector immune cells, that included NK cells and CD8^+^ T cells, that both contributed to enhancing tumor control but these studies did not look beyond these immune cells.^24^ In our more extensive analysis, we also observed a reduction in number of Tregs, known for their immunosuppressive potential, and an increased frequency of DCs for priming T cells and activating NK cells. Especially our finding of strongly increased amounts of CD103^+^ DCs with cross‐presenting potential in the tumor‐draining lymph nodes elucidates on the mechanism behind our observed anti‐tumor effects.

CD8^+^ T cells comprise an important compartment of the adaptive immune system with well‐established anti‐tumor effects. Upon T‐cell receptor activation, they produce effector cytokines like IFNγ and kill cancer cells in an antigen‐specific manner via the granzyme/perforin system.^31^, ^32^ Increased numbers of CD8^+^ T cell in cancer are therefore linked to better outcome and prognosis. With our combination strategy, we observed an increased number of CD8^+^ T cells which establishes a favorable tumor microenvironment for an anti‐cancer response. We found these cells were the most important player in our therapy models since depletion of these cells virtually abrogated the anti‐tumor response. Both CD40 and IL‐15 already as single modalities induced an increase in CD8^+^ T‐cell numbers but the combination therapy induced significantly higher intra‐tumoral infiltration of these effector cells. This might be due to certain chemokines since our unique gene expression analysis showed a higher expression of CXCR3 and its ligands CXCL9 and CXCL10 following combination treatment. These chemokines are known to be responsible for chemoattraction of both T cells and NK cells.^33^ Moreover, these two chemokines are correlated with increased survival and chemotherapeutic efficacy in PDAC in patients.^34^ Interestingly, CCL5 and CX3R1 were downregulated following combination therapy. However, the CCL5/CCR5 axis in PDAC has been shown to correlate with promotion of migration and invasiveness of the pancreatic cancer cells, and thus, downregulation could be actually beneficial in our models.^35^ Furthermore, the CX3CR1 axis is associated with early recurrence after surgery with poor patient prognosis.^36^ The activation marker CD69 was not shown to be upregulated on the CD8^+^ T cells. This may be due to the fact that tumors were harvested at day 8 post‐treatment and CD69 is considered to be an early activation marker which disappears after 4 days.^37^ Among the CD8^+^ T‐cell subsets, we found higher presence of CD8^+^ memory T cells which is linked to immune memory. IL‐15 has been described to be important for the induction and maintenance of these cells, and combining this modality with for instance PD‐L1 blockade might further increase the number of CD8^+^ memory T cells.^38^

Natural killer cells have become an increasingly important target for cancer immunotherapy since they have demonstrated to mediate successful and efficient anti‐tumor responses.^39^, ^40^ They play an important role in pancreatic cancer as we have shown here, and although depletion of NK cells did not have as drastic an effect on response to CD40 and IL‐15 combination as CD8^+^ T cells, it is likely that they still play an important role in this therapy given the increase in both their activation and numbers in the tumor. This is an important observation since a higher frequency of NK cells is clearly linked to better survival.^41^ IL‐15 is a strong stimulator of NK cells,^23^ and its effect is strongly increased by the addition of CD40 agonists that upregulate IL‐15Rα on DCs,^28^ which is necessary for trans‐presentation of IL‐15 to CD8^+^ T cells and NK cells.^20^ Moreover, since expression of IL‐12, IL‐15 and IL‐18 is upregulated, this may lead to formation of NK memory cells in the tumor microenvironment which needs to be further explored.^42^

Tregs have a high immune suppressive potential.^43^ When CD40 agonist was administered, we observed a significant reduction in the number of Tregs within the tumor, as confirmed by others in other solid tumor models.^26^, ^44^ The mechanism by which CD40 agonists cause Treg reduction still needs to be elucidated although one study indirectly points towards the blockage of interaction between myeloid‐derived suppressor cells and the Tregs.^45^ In addition, the highly increased CD8/Treg ratio following combination therapy was very encouraging since meta‐analysis showed that this is associated with improved overall survival in cancer patients^46^ and response to therapy.^47^, ^48^

Finally, reduction of Tregs in PDAC allows DCs to induce a more potent anti‐tumor immune response, largely mediated by CD8^+^ T cells.^49^ Our data demonstrated that DCs in general increased by 3‐fold in the tumor‐draining lymph nodes as a result of the combination therapy. Their importance in cancer has been extensively demonstrated as they function as the generals of our immune system by capturing tumor antigens and presenting them to T cells, thereby eliciting specific immune responses.^50^, ^51^ A very important subset of these DCs are the CD103^+^ DCs which are considered to reside in peripheral tissues. Upon activation, they migrate to the lymph nodes where they activate T cells by antigen presentation.^52^ Moreover, they have found to be the only APCs to transport intact antigens to tumor‐draining lymph nodes and prime tumor‐specific CD8^+^ T cells.^53^. Hence, our novel data showing a doubling in CD103^+^ DC in the tumor‐draining lymph nodes support the increased anti‐tumor responses we observed when CD40 was administered.

Today, more than 20 clinical trials using CD40 agonists like APX005M, Selicrelumab and CDX‐1140 are ongoing in cancer of which at least four are also including PDAC (NCT03214250, NCT02376699, NCT03329950 and NCT03193190). Furthermore, IL‐15 or pharmacologically enhanced IL‐15 superagonists like N‐803 are the subject of clinical trials with more than 50 ongoing studies of cancer of which at least four specifically focus on PDAC (NCT03329248, NCT03387098, NCT03586869 and NCT03136406). However, to our knowledge, no ongoing clinical trials use the combination presented here in this study. The data we presented here support a future clinical trial of IL‐15 combined with a CD40 agonist for PDAC, since we showed for the first time that this combination holds great potential for improved cancer treatment outcome.

## Methods

### Mice

Female C57BL/6j mice, age 6–8 weeks, were bred inhouse at the Peter MacCallum Cancer Centre or obtained from Jackson Laboratories (L'Arbresle, France). All mice were maintained at the Animal Core Facilities at the Peter MacCallum Cancer Centre or University of Antwerp. All animal procedures were conducted in accordance with, and approval of, the Animal Ethics Committee of the Peter MacCallum Cancer Centre under registration numbers E498 and E582 or University of Antwerp under registration number 2016‐30. All mice were housed in filter‐top cages enriched with houses and nesting material. Mice were checked on a daily base to inspect health and wellbeing. Mice were given a 7‐day adaptation period upon arrival before being included in experiments to reduce stress levels.

### Cell lines

Two different mouse PDAC cell lines were used. Panc02 is a chemically induced cell line while the KPC cell is derived from on an orthotopic tumor bearing the KRAS and p53 mutation. Both cell lines were cultured in DMEM cell culture medium (Life Technologies, Merelbeke, Belgium) supplemented with 10% FBS (Life Technologies) and 10 mm L‐Glutamine (Life Technologies). Cell lines were maintained at 37°C and 5% CO_2_. All cell lines were tested on a routine base for mycoplasma contamination. All cell lines were not passaged more than ten times between freeze and thawing and were only used in experiments between passage two and six.

### Tumor kinetics and survival

Prior to injection, Panc02 and KPC cells were harvested using TryplE (Life Technologies), washed thrice with sterile PBS and put through a 70‐µm cell strainer to assure single‐cell suspension without any contaminants. Next, mice were injected subcutaneously with either 0.5 × 10^6^ Panc02 or KPC cells suspended in 100 µL sterile PBS at the left abdominal flank. When tumors reached an average size of 25–35 mm^2^, mice were randomised based on tumor size and divided over four different treatment groups (day 0): (1) Isotype control; (2) IL‐15; (3) CD40 agonist; and (4) IL‐15 + CD40 agonist. Mice were given i.p. 2.5 µg IL‐15 (NCI) at days 0–3, 6–10 and 13–14. A mouse agonistic CD40 monoclonal antibody (Clone FGK‐45, BioXCell, obtained via Bio‐connect, Huissen, The Netherlands) or corresponding isotype control (Clone 2A3, BioXCell) was administered i.p. at days 0, 3, 7, 10 and 14 at a dosage of 12.5 µg per mouse for Panc02 or 200 µg (day 0) and 100 µg (days 3, 7, 10 and 14) for KPC tumors.

Tumor size was measured twice a week using a digital calliper (Chicago Brand, Medford, OR, USA). Tumor area was calculated using the formula length × width. Mice were sacrificed when a tumor size of 150 mm^2^ was reached or were stated as long‐term survivor when they survived 100 days post‐treatment without reaching the 150 mm^2^ threshold.

### Functional depletion experiments

For investigation of the role of different immune cell types, functional depletion experiments were carried out. Mice were given Panc02 or KPC tumors as described above. CD4^+^ and CD8^+^ T cells were depleted using 200 µg of αCD4 (Clone GK1.5, BioXCell) or αCD8 (Clone YTS 196.4, BioXCell) monoclonal antibodies, respectively, and NK cells were depleted using 25 µL of anti‐asialo‐GM1 (WAKO, Osaka, Japan) per mice. Mice were randomised into six different treatment groups: (1) Isotype control; (2) IL‐15 + αCD40; (3) IL‐15 + αCD40 + αCD4; (4) IL‐15 + αCD40 + αCD8; (5) IL‐15 + αCD40 + αNK; and (6) IL‐15 + αCD40 + αCD8 + αNK. Depletion antibodies or anti‐asioalo‐GM1 were given i.p. at days −1, 0, 3, 6, 10 and 14. Tumor kinetics and survival were measured as described above.

### RNA sequencing

For RNA sequencing, tumors were harvested on day 4 of the treatment scheme and transferred directly into RNAlater reagent (Qiagen, Antwerpen, Belgium). RNA was extracted using RNeasy mini plus kit or RNeasy midi kit depending on the sample weight. For removal of gDNA, RNAse‐free DNAse treatment was performed. Amount and quality of isolated RNA samples were assessed by fragment analysis. Library generation was performed using a Quantseq 3′ mRNA‐Seq library kit (Lexogen, Vienna, Austria) according to the manufacturer's instructions. NGS sequencing was performed on Illumina NextSeq. RNASeq data were processed using Seqliner RNASeq pipelines (v0.7; seqliner.org). The raw FASTQ sequenced samples were trimmed and aligned using Hisat v2 and quantified using HTSeq. In order to identify transcripts with increased or decreased expression, normalisation and differential expression analysis was performed with Limma‐Voom in R v3.3.1. On average, 16 073 genes were identified per sample and quantified. Full RNAseq was subsetted based on the NanoString nCounter Pan‐cancer Immune profiling panel of 770 genes. Principal component analyses (PCA), volcano plot and heatmaps were generated using R v3.6.1 using normalised log2 counts‐per‐million (CPM) values for relevant transcripts. Gene set enrichment analysis (GSEA) was performed on full normalised RNAseq data using the GSEA software package and public MSigDB v5.2 data sets. The mouse genes version of MSigDB was downloaded from http://bioinf.wehi.edu.au/software/MSigDB/. Parameters for GSEA analysis include the following: meandiv normalisation, max probe mode, signal to noise metric, gene set permutation, minimum gene set size of 15, and 1000 permutations. Molecular signature data sets used included the C2 curated gene set containing 4762 gene sets in version v5.2. We acknowledge our use of the gene set enrichment analysis, GSEA software, and Molecular Signature Database (MSigDB, Broad Institute, Waltham, MA, USA).^54^, ^55^

### Rechallenge experiments

To investigate induction of immune memory, rechallenge experiments were performed. Here, mice that were completely tumor‐free 100 days post‐start of treatment were re‐injected s.c. with either Panc02 or KPC cells at the contralateral flank of the primary tumor injection site. Tumor growth and survival were measured as described above.

### Characterisation of TIL and Tumor‐draining lymph nodes

To characterise tumor‐infiltrating lymphocytes (TIL), multicolour flow cytometry experiments were performed on KPC tumors and tumor‐draining lymph nodes from different treatment groups. Here, mice bearing KPC tumors were randomised and treated as described above. At day 8, mice were sacrificed and both tumor and tumor‐draining lymph node were removed during necropsy and weighed. Next, tumors were minced using scalpels followed by enzymatic digestion with digestion medium (RPMI 1640 + 10% FBS + 10 mm L‐glutamine + Collagenase D + DNAse‐I + Liberase) for 30 min at 37°C and 5% CO_2_ in a cell rocker. After digestion, all samples were washed with buffer (PBS + 2% BSA + 1 mm EDTA) and put through a 70‐µm cell strainer to obtain single‐cell suspension. Lymph nodes were dissociated mechanically, washed with FACS buffer and put through a 40‐µm cell strainer to obtain a single‐cell suspension.

Tumor single‐cell suspensions were stained with three different multicolour antibody panels, while the tumor‐draining lymph nodes were only stained with panel 3. Panel 1 consists of CD8‐FITC (Clone 53‐6.7, BD Biosciences, Erembodegem, Belgium), CD3‐PE (Clone 145‐2C11, Biolegend, Amsterdam, The Netherlands), CD4‐PercP‐Cy5.5 (Clone RM4‐5, Biolegend), CD69‐Pe‐Cy7 (H1.2F3, Biolegend), and NK1.1‐APC (Clone PK136, Biolegend); panel 2 of CD8‐BV421 (Clone 53‐6.7, BD Biosciences), CD25‐BV786 (Clone 3C7, BD Biosciences), CD4‐FITC (Clone GK1.5, Biolegend), CD3‐PE, CD44‐PerCP‐Cy5.5 (Clone IM7, Biolegend), CD62L‐Pe‐Cy7 (Clone MEL‐14, Biolegend), and FoxP3‐APC (Clone FJA‐16K, Biolegend); and panel 3 of CD8‐BV421 (Clone 53‐6.7, BD Biosciences), CD103‐BV786 (Clone M290, BD Biosciences), Ly6G‐FITC (Clone 1A8, Biolegend), CD11b‐PE (Clone M1/70, Biolegend), MHC‐II‐PE‐Cy7 (Clone M5/114.15.2, Biolegend), and CD11c‐APC (Clone N418, Biolegend). In all three panels, Live‐Dead Aqua (Life Technologies) was used as a viability staining and CD45.2‐APC‐Cy7 (Clone 104, Biolegend) was used to gate out leucocytes and not tumor cells. Prior to antibody staining, all cell suspensions were pretreated using Fc blocking antibody (Clone 2.4G2, BD Biosciences) to avoid aspecific binding of antibodies. Counting beads (Flow‐Count™ Fluorospheres, Beckman Coulter Life Sciences, Suarlée, Belgium) were added to allow calculation of absolute numbers which were also corrected for differences in tumor weight using the following formula:

$$\frac{\frac{\#\;\text{counted}\;\text{cells}}{\#\;\text{counted}\;\text{beads}}\;\times \;\#\;\text{added}\;\text{beads}}{\text{tumour}\;\text{weight}}$$

Fold changes, based on absolute counts and compared to the untreated control group, were calculated using the formula:

$$\frac{\text{absolute}\;\text{count}\;\text{per}\;g\;\text{tumour}\;\text{sample}}{\text{mean}\;\text{absolute}\;\text{count}\;\text{per}\;g\;\text{tumour}\;\text{control}}$$

All samples were analysed using a FACS Aria II flow cytometer.

### Statistics

Statistical differences in tumor kinetics between different treatment groups in different experiments were determined using a one‐way ANOVA with Bonferroni *post hoc* analysis. Differences in survival were analysed using a log‐rank test. To assess a difference between the amount of TIL in tumor by flow cytometry, a one‐way ANOVA with Bonferroni *post hoc* analysis was applied. Differences were considered to be significantly different if *P* < 0.05. Graphs were made using GraphPad v8.0 software (San Diego, CA, USA). Flow cytometry analysis was performed using FlowJo v10.6.3 (BD Biosciences). All statistical analyses were carried out in SPSS v26 (IBM, Brussels, Belgium).

## Conflict of interest

The authors declare no conflict of interest.

## Author contributions

**Jonas RM Van Audenaerde:** Conceptualization; Data curation; Formal analysis; Funding acquisition; Investigation; Methodology; Supervision; Validation; Visualization; Writing‐original draft. **Elly Marcq:** Data curation; Methodology; Writing‐review & editing. **Bianca von Scheidt:** Data curation; Methodology. **Ashleigh S Davey:** Data curation; Methodology; Writing‐review & editing. **Amanda J Oliver:** Data curation; Methodology. **Jorrit De Waele:** Data curation; Writing‐review & editing. **Delphine Quatannens:** Data curation. **Jinthe Van Loenhout:** Data curation. **Patrick P Pauwels:** Writing‐review & editing. **Geert Roeyen:** Conceptualization; Funding acquisition; Investigation; Methodology; Supervision; Writing‐review & editing. **Filip Lardon:** Funding acquisition; Writing‐review & editing. **Clare Y Slaney:** Conceptualization; Methodology; Supervision; Writing‐review & editing. **Marc Peeters:** Conceptualization; Funding acquisition; Methodology; Supervision; Writing‐review & editing. **Michael H Kershaw:** Conceptualization; Funding acquisition; Methodology; Supervision; Writing‐review & editing. **Phillip K Darcy:** Conceptualization; Funding acquisition; Methodology; Supervision; Writing‐review & editing. **Evelien LJM Smits:** Conceptualization; Funding acquisition; Investigation; Methodology; Project administration; Resources; Supervision; Writing‐review & editing.
