# Supplementary figures and images for: Novel combination immunotherapy for pancreatic cancer: potent anti‐tumor effects with CD40 agonist and interleukin‐15 treatment
Source: Clin Transl Immunology. 2020 Aug 15;9(8):e1165. doi: 10.1002/cti2.1165 (PMC7428816; doi:10.1002/cti2.1165)

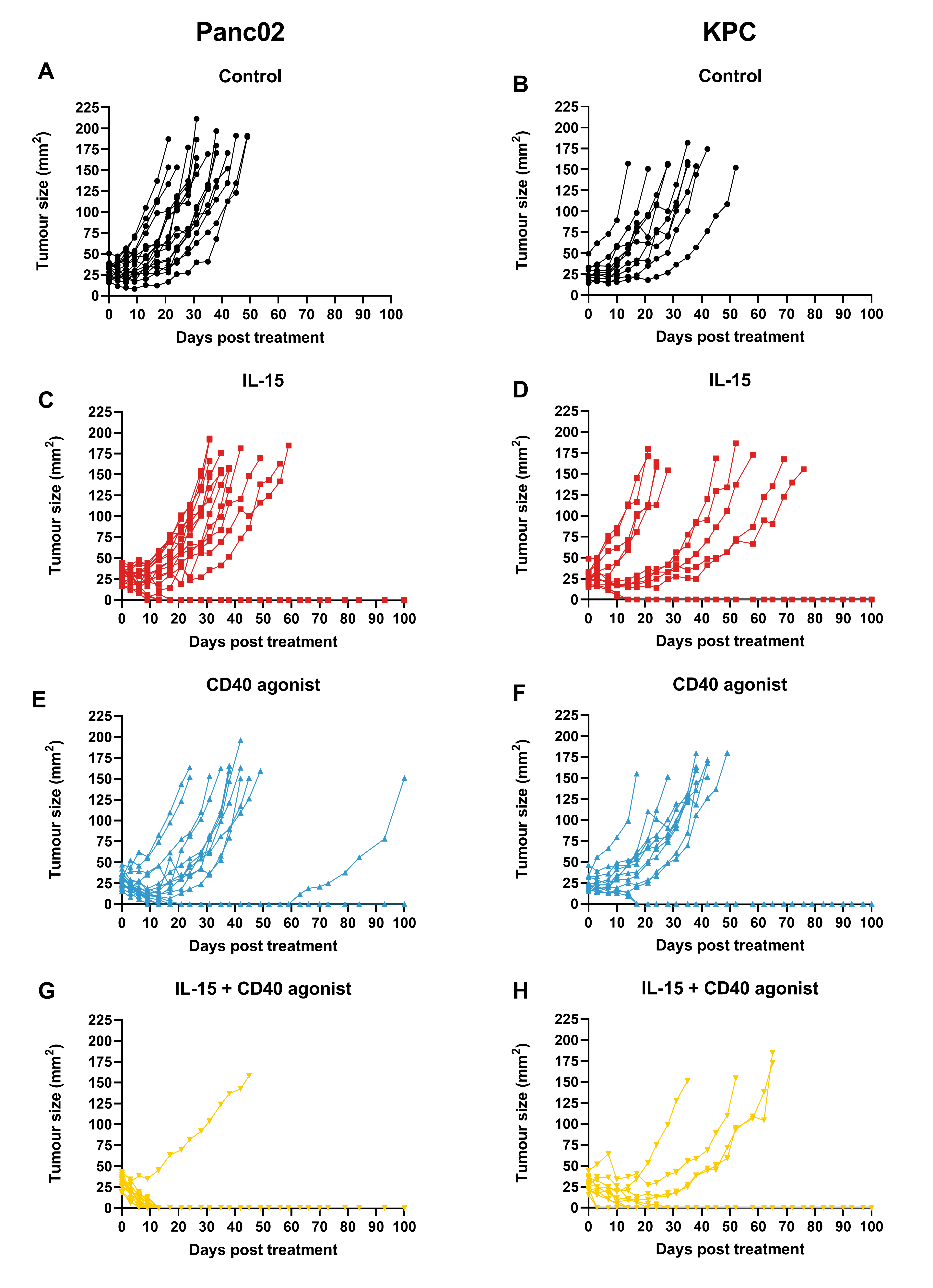

Supplement: Supplementary file 1 [file CTI2-9-e1165-s001.tif]

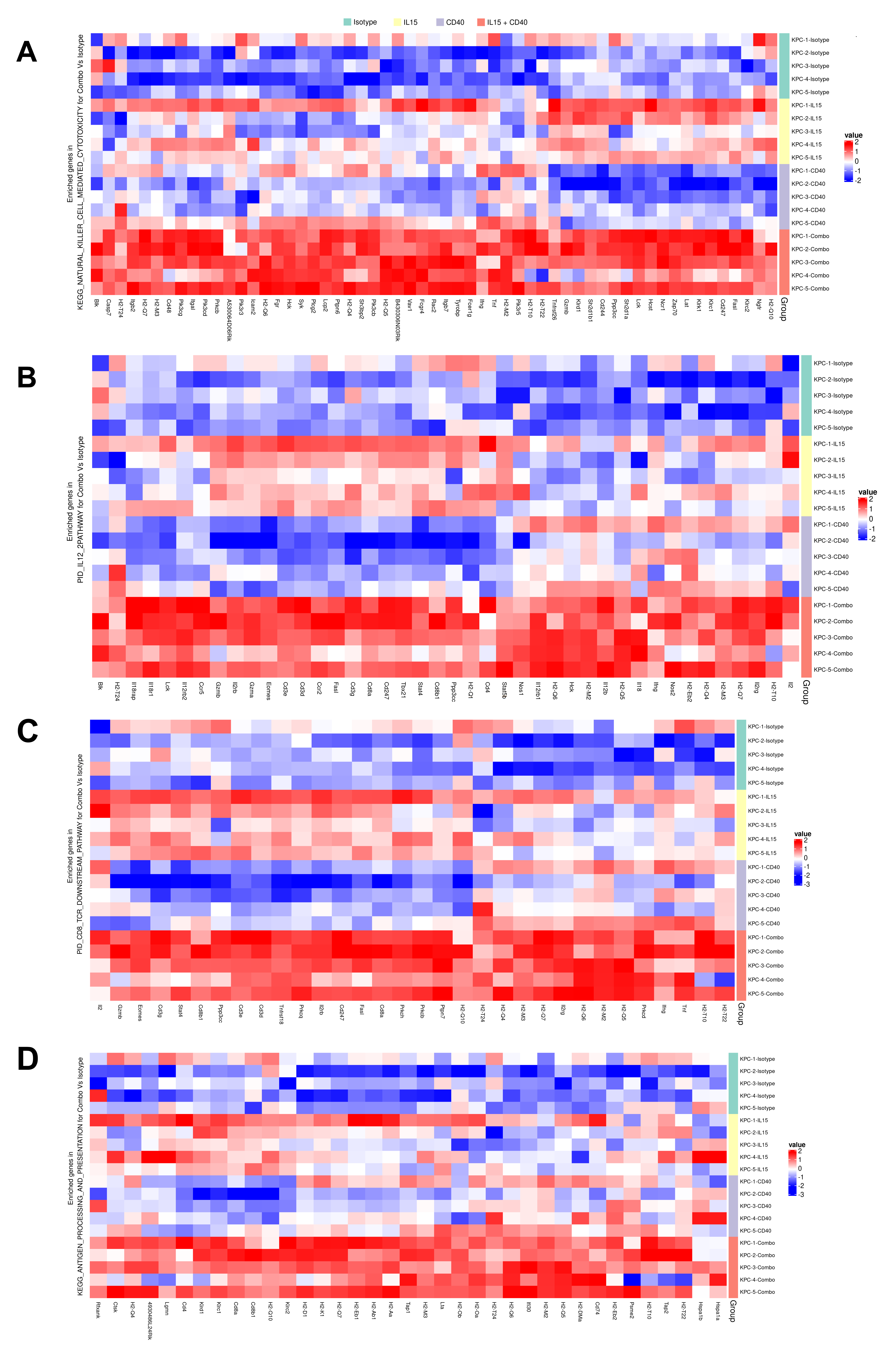

Supplement: Supplementary file 2 [file CTI2-9-e1165-s002.tif]

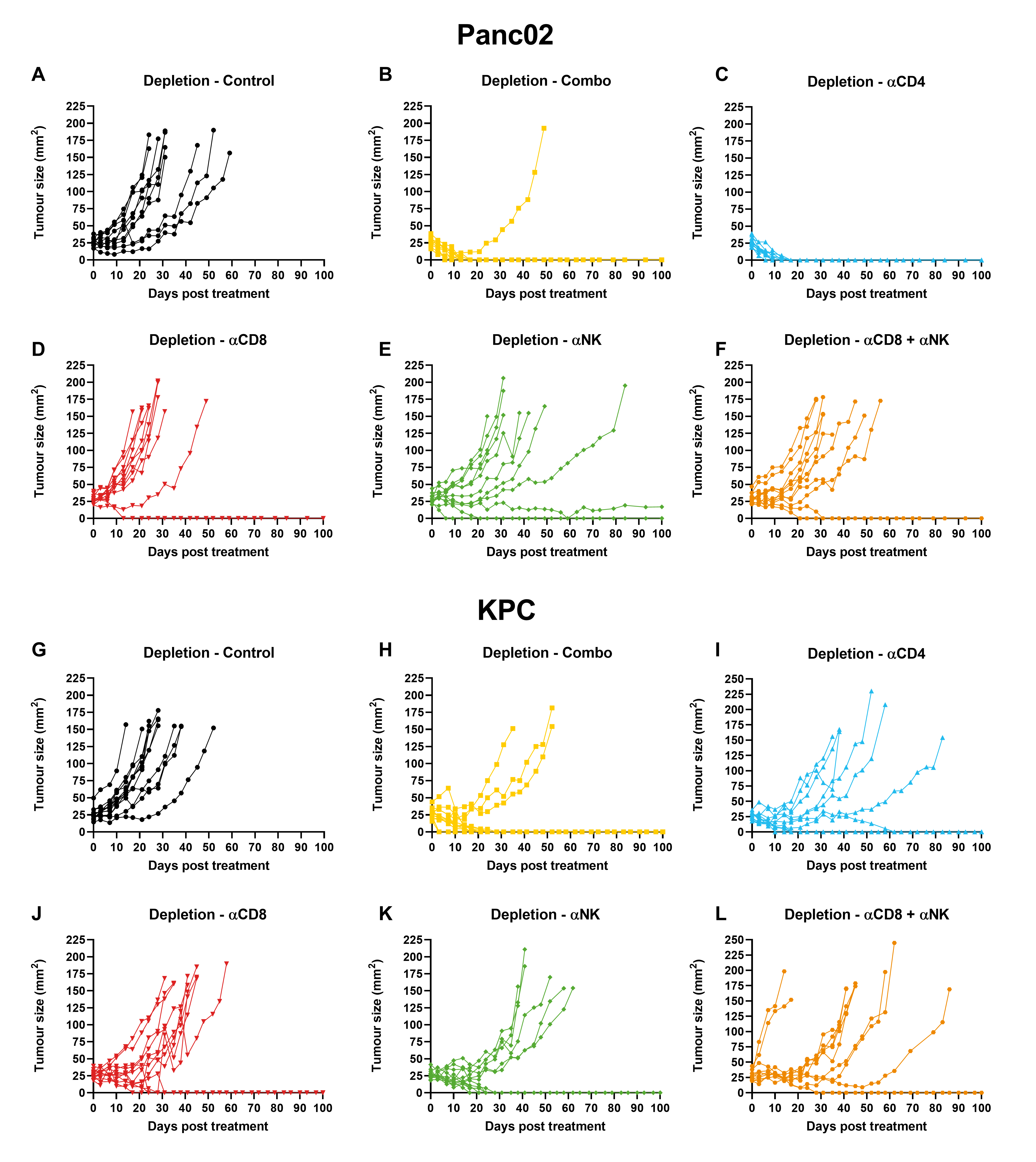

Supplement: Supplementary file 3 [file CTI2-9-e1165-s003.tif]

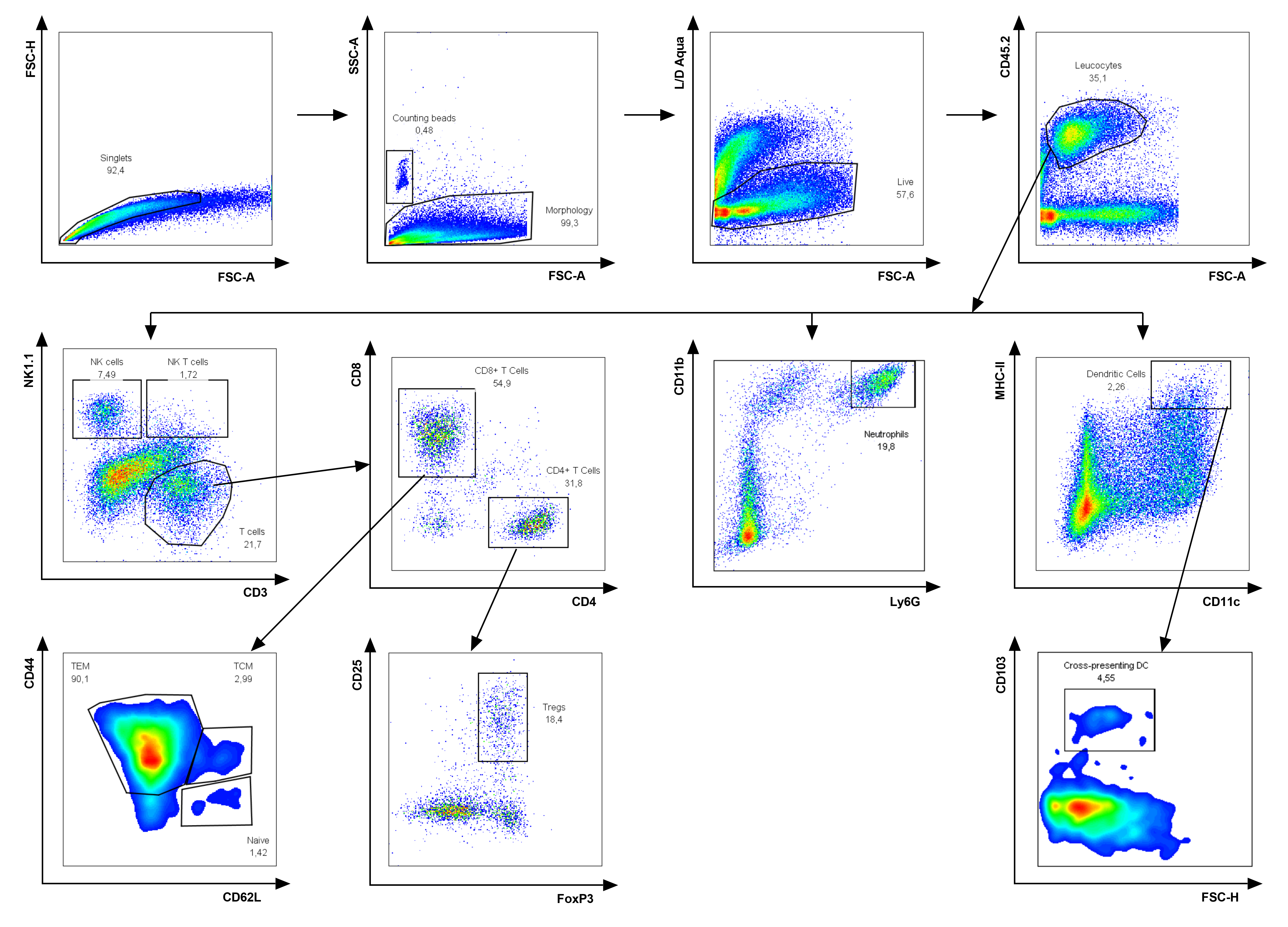

Supplement: Supplementary file 4 [file CTI2-9-e1165-s004.tif]
