# Supplementary material for: Novel combination immunotherapy for pancreatic cancer: potent anti‐tumor effects with CD40 agonist and interleukin‐15 treatment
Source: Clin Transl Immunology. 2020 Aug 15;9(8):e1165. doi: 10.1002/cti2.1165 (PMC7428816; doi:10.1002/cti2.1165)
Supplement: Supplementary file 5 [file CTI2-9-e1165-s005.docx]

**Supplementary Figure 1. Tumor growth kinetics of survival experiment**

C57BL/6j mice were injected with either 0.5x10^6^ Panc02 or KPC cells subcutaneously. When tumors reached a size of 25-35 mm², mice were randomised and treated with isotype control, IL-15, CD40 agonist or IL-15 + CD40 agonist. Tumor growths kinetics are depicted for each mouse per treatment group for either Panc02 (n = 17-18 / group) (**a**, **c**, **e**, **g**) or KPC (n = 10-12 / group) (**b**, **d**, **f**, **h**).

**Supplementary Figure 2. Heatmaps of GSEA enriched pathways**

KPC tumors were harvested on day 4 of the treatment schedule for subsequent RNA isolation and sequencing. Heatmaps of GSEA enriched gene sets depicted in Fig 2 are shown with differentially expressed genes in the **(a)** KEGG Natural Killer Mediated Cytotoxicity, **(b)** PID IL-12/2 pathway, **(c)** PID CD8^+^ TCR Downstream and **(d)** KEGG Antigen Processing and Presentation. n = 5 tumors/group.

**Supplementary Figure 3. Tumor growth kinetics of immune depletion experiment**

C57BL/6j mice were injected with either 0.5x10^6^ Panc02 or KPC cells subcutaneously. When tumors reached a size of 25-35 mm², mice were randomised and treated with isotype control or the IL-15 + CD40 agonist combination regimen alone or with depleting antibodies against CD4, CD8, asialo-GM1 (NK cell depletion). Tumor growths kinetics are depicted for each mouse per treatment group for either Panc02 (n = 9-11 / group) **(a - f)** or KPC (n = 11-13 / group) **(g - l)**.

**Supplementary Figure 4. Flow cytometry gating strategy**

Gating strategy applied in figure 4. Plots are representative from 3 independent experiments.
